# Comparison of endoscopic versus CT assessment of stone-free status after percutaneous nephrolithotomy (PCNL)

**DOI:** 10.1007/s00240-023-01495-7

**Published:** 2023-10-06

**Authors:** F. O. Hartung, K. J. Müller, J. Herrmann, B. Grüne, M. S. Michel, M. C. Rassweiler-Seyfried

**Affiliations:** Department of Urology and Urologic Surgery, University Medical Center Mannheim, University of Heidelberg, Theodor-Kutzer-Ufer 1-3, 68167 Mannheim, Germany

**Keywords:** Stone-free rate, Percutaneous nephrolithotomy, PCNL, Low-dose CT

## Abstract

This study is aimed to determine whether postoperative low dose computed tomography (LDCT) imaging is necessary after percutaneous nephrolithotomy (PCNL), or the surgeon's intraoperative assessment of residual fragments (RF) is sufficient and avoidance of postoperative imaging with reduction of radiation exposure can be achieved. Data of all 610 patients who underwent PCNL in prone position in our institution from February 2009 to September 2020 was collected. Parameters such as age, gender, BMI, ASA-Classification, stone related parameters and the surgeon’s assessment of stone-free status were analyzed. The LDCT performed postoperatively was compared to the intraoperative assessment of the surgeon regarding RF. The mean age of patients was 52.82 years; the mean BMI was 28.18 kg/m^2^. In 418 cases, the surgeon made a clear statement about the presence of RF and postoperative LDCT was carried out. The discrepancy between the two methods (surgeon´s assessment vs. LDCT) was significant at p < 0.0001. The sensitivity, specificity, positive and negative predictive value of the surgeon when assessing RF were 24.05%, 99.45%, 98.28% and 50%. Stone free rate (SFR) after primary PCNL was 45.57%. The overall SFR at discharge was 96.23%. Although the surgeon´s assessment of RF was reliable, postoperative LDCT imaging should still be performed if endoscopic stone clearance is suspected due to the high false negative rate and the low negative predictive value. The optimal timing of postoperative imaging following PCNL remains unclear.

## Introduction

Urolithiasis is one of the most common diseases worldwide with increasing prevalence and incidence in the recent decades [1–3]. Consequently, diagnosis, treatment and prevention of stone recurrence are associated with significant costs [4]. Since the late 1980s, the percutaneous nephrolithotomy (PCNL) technique has been regarded as the standard therapy for large kidney stones with very good stone-free rates (SFR) [5, 6]. According to the European Association of Urology (EAU) guidelines, PCNL is the first-line therapy for kidney stones > 20 mm, whereas kidney stones between 10 and 20 mm can be treated by extracorporeal shock wave lithotripsy (ESWL), ureterorenoscopy (URS) and PCNL [7]. The objective of PCNL is to achieve stone-free-status (SFS) to minimize the risk of future stone-related events and accompanying surgeries and interventions [8]. However, residual fragments (RF) after PCNL are common [9]. To detect RF, imaging methods such as kidney-ureter-bladder radiography (KUB), sonography or low dose computed tomography (LDCT) can be used after the first intervention [9, 10]. Compared to KUB and sonography, LDCT might expose patients to higher doses of radiation but can be performed quickly, does not require contrast agent administration, and detects RF of all sizes with high sensitivity [11, 12]. Only few studies have investigated the correlation between intraoperative SFS defined by the surgeon compared with postoperative LDCT [13, 14].

The aim of this study is to investigate how reliable the surgeon's intraoperative assessment of SFS is and to clarify whether an additional postoperative LDCT is necessary.

## Materials and methods

### Study design and parameters

Data was retrieved retrospectively from all 716 patients who received a percutaneous stone removal from 18.02.2009 to 21.09.2020 at the Department of Urology and Urological Surgery at the University Medical Center Mannheim (institutional review board approval 2020-837R).

**Fig. 1:**
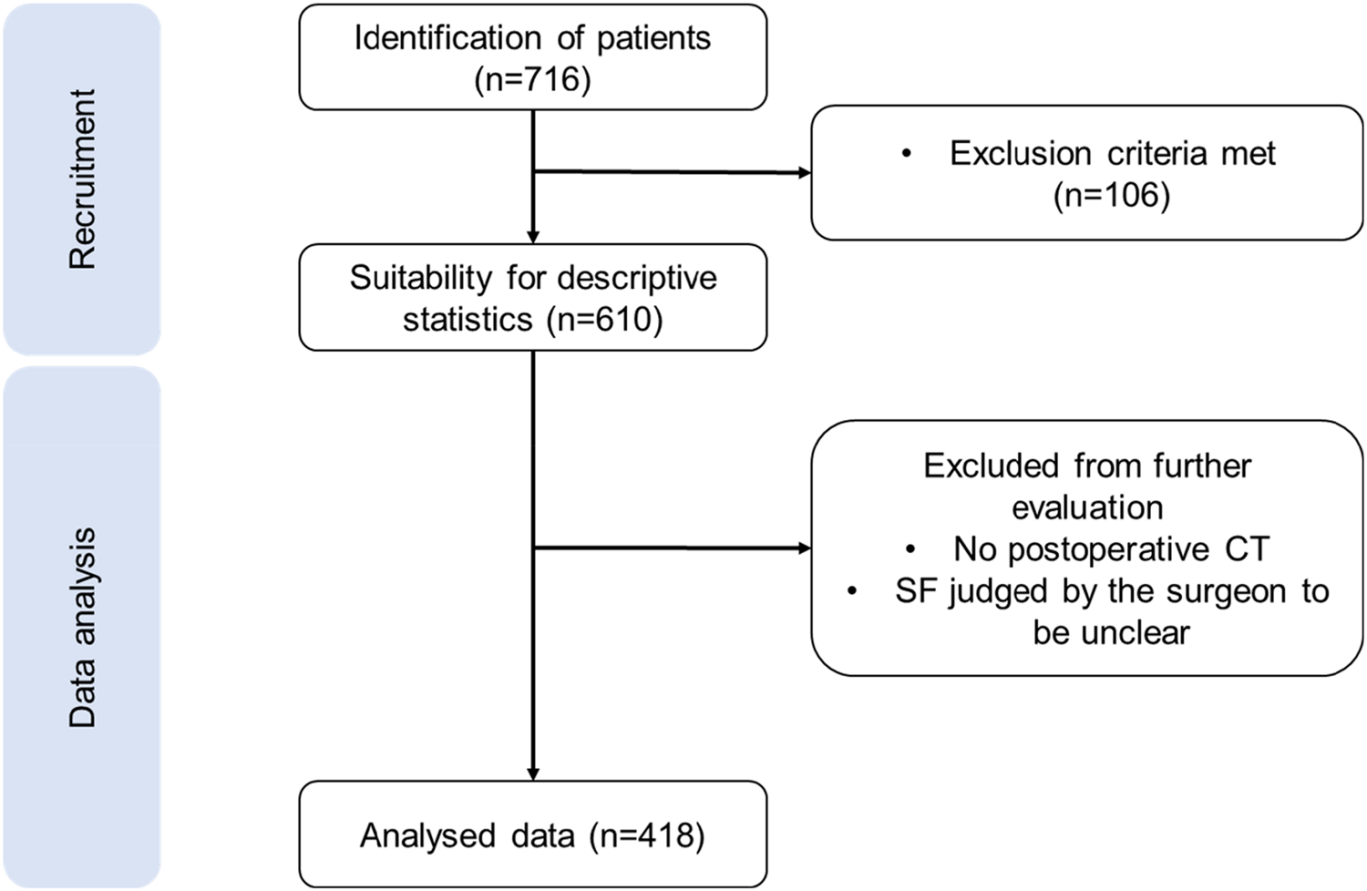
Selection chart

We evaluated preoperative parameters such as age at surgery, body mass index (BMI), gender, American Society of Anesthesiologists (ASA)-Classification, preoperative via CT determined stone characteristics (side, quantity, intrarenal localization, size), the Guy´s stone score (GSS) as well as following intraoperative and postoperative parameters: positioning of the patient, bore size, operating time, surgeon’s intraoperative assessment of SFS, postoperative CT-graphical assessment of SFS after PCNL, reintervention rate and intraoperative assessment of RF size (> 4 mm)/no stone detection) in case of an additional intervention. If, according to postoperative imaging, RF were still present, their location, amount and size were determined. Regarding the size, a distinction was made between significant (> 4 mm) and non-significant (≤ 4 mm) RF. This classification was based on a study by Hubner et al. from 1993 analyzing the incidence of spontaneous stone passage (SPP) relating both stone size and location [15]. The following exclusion criteria were defined: other than prone position, complementary surgery such as URS, missing surgical record, age < 16 years (Fig. 1). PCNL other than in prone position was excluded from this study to provide uniform interventions.

Out of our collected database, following variables were used for statistical analysis: stone-free-status assessed by the surgeon and according to the postoperative LDCT, size of the RF (< 4 mm), stone free rate after the first procedure and on dismissal.

Only cases with a postoperative CT-graphic classification of RF into significant (> 4 mm) and not significant (≤ 4 mm), as well as an intraoperative assessment by the surgeon into relevant RF (> 4 mm) and no stone detection were considered. No patient in this study had spina bifida or spinal injury and only complete staghorn stones were rated as GSS IV.

### Procedures

PCNL was performed by experienced surgeons with at least 50 cases, or under the supervision of an experienced surgeon. All cases were performed in prone position and under general anesthesia. Renal puncture was performed under ultrasonographic and fluoroscopic guidance. Different nephroscope (Karl Storz, Tuttlingen, Germany) sizes from 4,8 Ch to 32 Ch were used. Standard PCNL had a bore size of 24–32 Ch, mini-PCNL of 14–22 Ch, ultra-mini-PCNL of 11–13 Ch and micro-PCNL of < 11 Ch. Stones were removed in toto or after laser, ultrasonographic or pneumatic fragmentation. SFR was judged at the end of the procedure via fluoroscopy or flexible antegrade nephroscopy trough the tract by the surgeon and based on his preference a nephrostomy tube or a ureteral stent was placed. Postoperative imaging was routinely performed on the first postoperative day and was evaluated by the radiologists. If the radiologist described RF without details regarding the size, the size was labeled as “not specified” in this study. Papilla calcifications were not considered as RF. Regarding the operation time, a distinction was made between a longer duration of surgery (≥ 110 min) and a shorter duration of surgery (< 110 min). This cut-off resulted from the median of the statistical analysis.

### Statistical analysis

For external data analysis, the Data Export Tool was used to export the collected data from the REDCap system to an Excel database. The statistical analysis was performed with JMP^®^ version°14 (SAS Institute Inc., Cary, NC).

All parameters are presented as mean ± standard deviation (SD) in case of continuous data. For binary parameters, absolute and relative frequencies are given.

The McNemar test was used to compare the nominally scaled variables of the intraoperative and CT graphical assessment of SFS. A p-value < 0.05 was considered statistically significant. In addition, general values of sensitivity, specificity and predictive values were determined. The predictive values were calculated based on the prevalence determined in the CT.

For the analysis of influencing factors for a discordance between the surgeon´s and postoperative CT graphic assessment of SFS, a group comparison was carried out. Various factors were compared with the Pearson Chi-square test between cases of concordant and discordant stone-free status.

A univariable and a multivariable logistic regression analysis was performed to investigate the influence of various factors on the postoperative RF rate. In both univariable and multivariable analysis, an odds ratio (OR) was used as a measure of risk and its 95% confidence interval (95% CI).

## Results

The descriptive analysis included a total of 610 PCNL interventions performed between February 2009 and September 2020 (Fig. 1). Patient’s characteristics, as well as intraoperative and postoperative patient´s cohort parameters, are presented in Table 1. In 126 cases, no postoperative imaging was performed because additional surgical interventions were planned or SFS was diagnosed by the surgeon and a CT was not necessary (e.g. in recurrent stone formers) or an ordered CT was not available or a CT was performed 3–4 weeks postoperatively in an outpatient setting.

**Table 1:** Patient´s characteristics and perioperative parameters

Variables	
Patient´s characteristics	
Age (in years), Mean ± SD	52.82 ± 14.90
Gender (n = 610), n (%)	
Male	378 (61.97)
Female	232 (38,03)
BMI (kg/m^2^), (n = 460), Mean ± SD	28.18 ± 5.91
Side of stone localisation (n = 610), n (%)	
Right	254 (41.64)
Left	354 (58.03)
Bilateral	2 (0.33)
Number of stones (n = 610), n (%)	
1	311 (50.98)
2	102 (16.72)
3	59 (9.67)
4	26 (4.26)
≥ 5	102 (16.72)
Not specified	10 (1.64)
Stone localisation (n = 610), n (%)	
Upper calyx	131 (21.48)
Middle calyx	119 (19.51)
Lower calyx	330 (54.10)
Renal pelvis	375 (61.48)
Staghorn stone	136 (22.30)
Ureter	71 (11.64)
Not specified	9 (1.48)
Maximum stone size (diameter in mm), Mean ± SD	
Upper calyx group (n = 72)	14.67 ± 12.68
Middle calyx group (n = 71)	12.86 ± 9.16
Lower calyx group (n = 260)	13.04 ± 7.89
Renal pelvis (n = 308)	18.80 ± 10.93
Staghorn stone (n = 81)	28.06 ± 15.88
Ureters (n = 50)	10.42 ± 7.99
Not specified (n = 5)	12.00 ± 7.31
Guy´s stone score (n = 610), n (%)	
0	1 (0.16)
I	187 (30.66)
II	234 (38.36)
III	170 (27.87)
IV	18 (2.95)
ASA-Classification (n = 610), n (%)	
I	130 (21.31)
II	352 (57.70)
III	117 (19.18)
IV	11 (1.80)
Intraoperative parameters	
Bore size (n = 572), n (%)	
Standard PCNL	330 (57.69)
Mini-PCNL	240 (41.96)
Ultra-mini-PCNL	1 (0.17)
Micro-PCNL	1 (0.17)
Fragmentation (n = 610), n (%)	
No fragmentation	129 (21.15)
Laser	272 (44.59)
Pneumatic	5 (0.82)
Ultrasound	239 (39.18)
Not specified	19 (3.12)
Operation time (min), (n = 591), Mean ± SD	115.93 ± 43.57
SFS judged by surgeon (n = 610), n (%)	
RF	153 (25.08)
No RF	445 (72.95)
Not specified	12 (1.97)
Postoperative parameters	
Length of hospital stay (days), Mean ± SD	4,61 ± 3,46
Postoperative imaging (n = 484), n (%)	
LDCT	429 (88,64)
Intraoperative Dyna-CT	1 (0,21)
Sonography	1 (0,21)
X-ray	76 (15,70)
Other	2 (0,41)
Size of RF (in mm) (n = 348), n (%)	
> 4mm	141 (40,52)
≤ 4mm	114 (32,76)
Not specified	93 (26,72)

### Analysis: Intraoperative versus CT graphic assessment of SFS

In 418 cases, the surgeon made a clear statement about the presence of RF and a postoperative LDCT was carried out. RF were detected more frequently with postoperative LDCT (n = 237) than the surgeon judged intraoperatively (n = 58), resulting in a significant difference between the two methods in the McNemar test (p < 0.0001). Conformity between both methods (surgeon vs. LDCT) is low with a kappa index of 0.21.

**Fig. 2:**
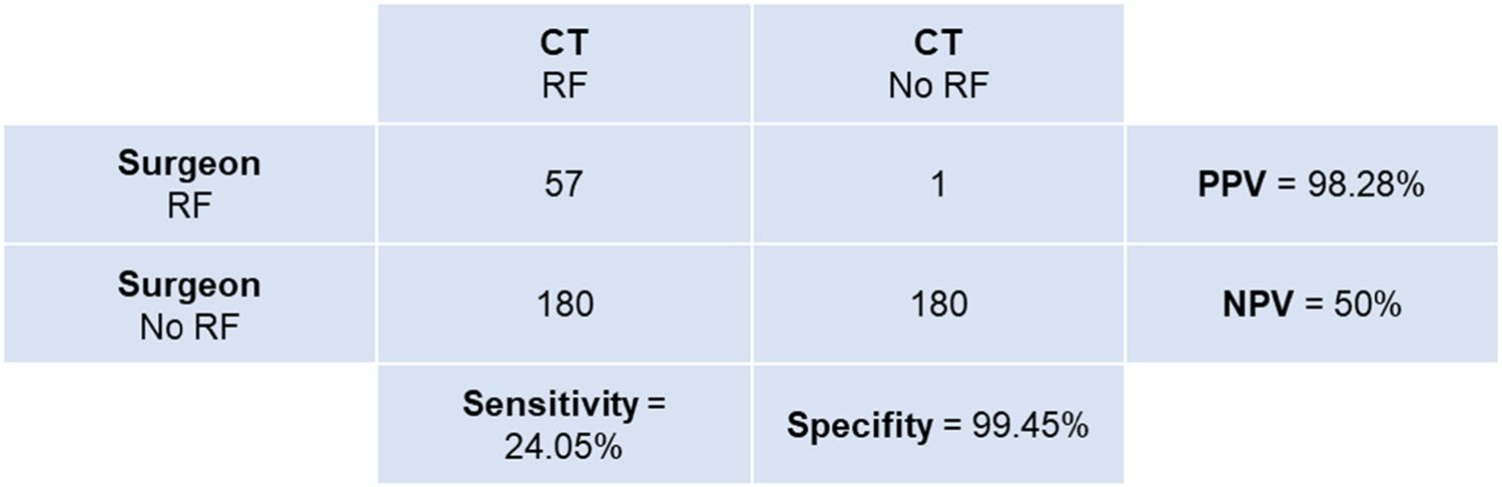
McNemar Test comparing intraoperative and CT graphic assessment of SFS

The sensitivity, specificity, positive and negative predictive value (PPV/NPV) of the surgeon in the assessment of RF is shown in Fig. 2. Out of the 180 cases wrongly judged by the surgeon to be stone-free, 53.33% had RF size ≤ 4 mm, 38.89% had RF size > 4 mm and in 7.78% no information was given regarding RF size.

### Stone free rate after initial PCNL and at discharge

SFR after the initial PCNL was 45.57% (n = 278). In 22 cases (3.61%) no RF was detected in an additional procedure. Including the RF ≤ 4 mm without the need of an additional intervention (n = 72, 11.80%), this resulted in a total SFR of 57.37% after primary PCNL.

The overall SFR at discharge was 96.23% (n = 587) after secondary procedures such as URS or another PCNL in the same inpatient stay.

## Discussion

A study by Harraz et al. from 2017 analyzed data from 306 patients undergoing PCNL which showed that the sensitivity of the intraoperative assessment of SFS by the surgeon was 49.6% and the PPV was 92.8% (specificity: 97.1% NPV: 72%) [16]. In 2018 Nevo et al. analysed data from 312 patients undergoing PCNL. In this study RF < 4 mm were additionally evaluated as stone-free by the surgeon as well as by postoperative imaging. The NPV of the surgeon’s assessment for SFS was 100% (sensitivity: 100%, specificity: 12.5%, PPV: 75%) [14]. In another study, conducted by Jannello et al. in 2022, the match rate between intraoperative SFS and CT-based SFS was determined after vacuum-assisted PCNL [13]. In this study the surgeon was able to accurately predict SFS in 77% of cases. Our results support the high false negative rate and low NPV, which in this study was 50%. Patients with kidney abnormalities and large stones were included in our study, and stones were defined as RF regardless of their size. In addition, it must be noted that in the context of early postoperative imaging, just as with our study, false-positive CT findings e.g. stone dust are possible, which can influence the NPV. Then again if CT imaging is performed 3 months postoperatively, as in the study by Jannello et al. RF can spontaneously go off or even increase in size in the time between surgery and imaging. The EAU recently updated its guidelines and recommends that regular imaging (plain film X-ray and/or sonography) should take place up to 60 months after treatment in case of RF, independent of their size. CT should be considered if patient is symptomatic or if intervention is planned [7]. Regarding RF and aftercare, it can be mentioned at this point that there is only limited data regarding spontaneous stone passage (SSP) according to stone size in case of a RF outlet into the ureter. A study by Yallappa et al. from 2018 analysed 6600 patients with ureteral stones and showed that SPP was reported for 68% of distal ureteral stones, 58% of mid ureteral stones and 49% of upper ureteral stones. Considering stone size almost 75% of stones < 5 mm and 62% of stones > 5 mm passed spontaneously in this study [17]. A more recent multi-center study by Shah et al. from 2019 reported SPP for ureteral stones ≤ 5 mm to be 89% if localized in the distal ureter, 80% if localized in the middle ureter and 71% if localized in the upper ureter [18].

In their studies, Harraz et al. Nevo et al. and Jannello et al. also addressed predictors for an accurate surgeon´s assessment regarding RF. Harraz et al. were able to show that a low GSS had the highest predictive power for a correct surgeon´s assessment for the absence of RF in a multivariate analysis. Nevo et al. showed that the surgeon had missed RF more frequently in patients with increasing stone size and quantity. Jannello et al. were able to identify a larger stone volume, a higher rate of multiple stones, a higher rate of several calyx-groups affected by stones, and a higher rate of GSS II degrees as independent predictors of a discordant stone-free status. Our results confirm the results of Jannello et al. regarding the number of stones and the affected groups of calyxes as well as the results of Nevo et al. regarding the number of stones.

Our multivariable logistic regression analysis shows that an operation time of ≥ 110 min, a GSS degree ≥ II and an initial maximum stone size ≥ 15 mm was associated with a higher rate of postoperative RF. ASA-Classification and bore size was not associated with a higher rate of postoperative RF in our multivariable logistic regression analysis.

The results of this study show that despite the surgeon's ability to detect RF as such after PCNL, postoperative imaging is still necessary to avoid missing RF. Instead of postoperative CT, intraoperative imaging with low radiation exposure, which is sensitive for the assessment of RF, could be performed. The surgeon’s intraoperative assessment of stone-free status would thus be supported by imaging and an improvement in the SFR could be achieved.

The Uro Dyna-CT (ArtisZee Siemens Healthcare Sector, Erlangen, Germany) already exists for the intraoperative detection of RF. It causes lower radiation exposure at CT-like image quality when identifying kidney stones compared to standard CT [19]. Meister et al. showed that RF can be detected using the Uro Dyna-CT and their size can be measured intraoperatively with high accuracy. In 2017 Vincentini et al. reported a case in which Uro Dyna-CT was performed intraoperatively with simultaneous removal of fragments residual stones that were not found by digital fluoroscopy and flexible nephroscopy at the end of surgery [20]. However, installing this system is costly and not widely available [10].

At this point, the possibility of endoscopic combined intrarenal surgery (ECIRS) should also be considered, which, according to a meta-analysis by Widyokirono et al. [21], is an effective and safe treatment particularly for large and complex nephrolithiasis, with significantly higher one-step SFR, a lower necessity for auxiliary procedures, and a lower complication rate compared with PCNL.

This study has limitations due the retrospective design and the unicentric location. The preoperatively collected data on the affected side and the stone location did only provide information about the stones that were planned to be removed as part of the PCNL procedure. Furthermore, a patient could have been treated several times with PCNL in the context of different stone episodes, which leads to limitations in the descriptive statistics of patient history data. Furthermore, nephroscopy was performed anterograde through the tract which may have less adequate access to all calyces as opposed to a supine approach with a flexible ureteroscope.

## Conclusions

The surgeon’s intraoperative evaluation on its own is not reliable in evaluating the absence RF. We recommend performing LDCT after PCNL to ensure a stone free status. LDCT may be omitted, if the surgeon reports intraoperative RF. Measures to improve SFR after the first intervention should be taken such as antegrade and retrograde nephroscopy and intraoperative CT imaging if available.
